# 4353 The Role of BCL2 Mediated Calcium Signaling on Leukemia Stem Cell Metabolism

**DOI:** 10.1017/cts.2020.98

**Published:** 2020-07-29

**Authors:** Anagha Inguva, Shanshan Pei, Maria Amaya, Brett Stevens, Courtney Jones, Daniel Pollyea, Craig Jordan

**Affiliations:** 1 University of Colorado at Denver; 2 University of Colorado

## Abstract

OBJECTIVES/GOALS: The objective of this study is to define the molecular mechanisms that control survival of malignant stem cells in acute myeloid leukemia (AML). Leukemia stem cells (LSCs) are not effectively eradicated by standard treatment and lead to resistance and relapse, which contribute to poor survival rates. METHODS/STUDY POPULATION: The recently FDA approved venetoclax, a BCL2 inhibitor, with azacitidine, a hypomethylating agent leads to a 70% response rate in AML patients. Analysis of patients treated with this regimen showed direct targeting of LSCs. BCL2 has a non-canonical function in regulation of intracellular calcium. To determine how BCL2 mediated calcium signaling plays a role in LSC biology, we used LSCs isolated from venetoclax/azacitidine (ven/aza) sensitive and resistant patient samples to measure expression of calcium channels via RNA seq. BIO-ID, siRNA, flow cytometry, seahorse assays, calcium measurements and colony assays were used to determine the effects of calcium channel perturbation on LSC biology. RESULTS/ANTICIPATED RESULTS: BCL2 inhibition leads to decreased OXPHOS activity in primary AML specimens. BIO-ID studies revealed cation/metal ion transporters, ER membrane proteins and ER membrane organization as top enriched pathways interacting with BCL2. RNA-seq data showed increased expression of genes involved in calcium influx into the ER in ven/aza sensitive LSCs and increased expression of genes involved in calcium efflux from the ER in ven/aza resistant samples. Ven/Aza resistant LSCs have increased mitochondrial calcium content, consistent with their increased OXPHOS activity as calcium is required for OXPHOS. Perturbation of these channels leads to decreased OXPHOS activity and decreased viability in LSCs. DISCUSSION/SIGNIFICANCE OF IMPACT: We postulate that a deeper understanding of the mechanisms behind ven/aza targeting of LSCs will lead to the development of novel therapies for patients who do not respond to ven/aza. Our data show targeting intracellular calcium signaling could be a viable therapeutic strategy for AML patients.

